# Parasites in Sewage: Legal Requirements and Diagnostic Tools

**DOI:** 10.3390/pathogens14010086

**Published:** 2025-01-16

**Authors:** Oliwia Obuch-Woszczatyńska, Klaudia Bylińska, Małgorzata Krzyżowska, Karol Korzekwa, Piotr Bąska

**Affiliations:** 1 Laboratory of Parasitology, Military Institute of Hygiene and Epidemiology, 01-001 Warsaw, Poland; oliwia.obuch@wihe.pl (O.O.-W.); klaudia.bylinska@wihe.pl (K.B.); 2 Division of Pharmacology and Toxicology, Department of Preclinical Sciences, Institute of Veterinary Medicine, Warsaw University of Life Sciences, 02-786 Warsaw, Poland; 3 Department of Medical and Environmental Microbiology, Military Institute of Hygiene and Epidemiology, 01-001 Warsaw, Poland; malgorzata.krzyzowska@wihe.pl (M.K.); karol.korzekwa@wihe.pl (K.K.)

**Keywords:** parasites, sewages, invasiveness, roundworms, Ascaridae, wastewater

## Abstract

Despite the vast amount of water on Earth, only a small percent is suitable for consumption, and these resources are diminishing. Moreover, water resources are unevenly distributed, leading to significant disparities in access to drinking water between countries and populations. Increasing consumption and the expanding human population necessitate the development of novel wastewater treatment technologies and the use of water treatment byproducts in other areas, such as fertilisers. However, water treatment sludge often cannot be used to enhance crop production due to the presence of parasite eggs, particularly from roundworms (Ascaridae family), which are resistant to environmental factors and can pose a threat for several years. Legislation prohibits the use of sludge containing parasite eggs as fertiliser. In some cases, water may not contain parasite eggs but larvae, which require different detection methods. Additionally, the presence of eggs does not necessarily indicate danger since they may lose infectivity due to prolonged storage or exposure to chemical compounds in the sewage. This paper reviews European Union regulations on wastewater treatment, the selected parasitic diseases related to the presence of parasites in wastewater, the spectrum of detection methods, and highlights differences in viability and invasiveness, which is intended to draw attention to the need to determine both biological properties of parasites.

## 1. Introduction

Following the European Council Directive of 21 May 1991 [1], wastewater is understood as water used during human activities for domestic or economic purposes. Additionally, the term wastewater includes, among others, liquid animal waste, leachate, water from the cooling cycles of power plants or cogeneration plants, water from mine drainage, and water used and discharged from fish farming or breeding facilities. Furthermore, Asthana et al. [2] define wastewater as liquid or waterborne waste removed from homes, industrial and commercial establishments, and various institutions, along with surface water, groundwater, and rainwater. In this context, wastewater includes faeces, washing water, and everything that flows from homes to the sewer system [2]. Others define wastewater as water which can no longer be used or as water, the disposal of which is more cost-effective [3].

According to the European Council Directive of 21 May 1991 [1], concerning urban wastewater treatment, three types of wastewater can be distinguished: domestic, industrial, and municipal. These types of wastewater are categorised based on their origin and chemical composition. Domestic wastewater comprises waste from residential and public utility buildings from the human metabolism and household activities. This type of wastewater includes faeces, food residues, and various detergents. Industrial wastewater is defined as all wastewater (excluding domestic wastewater, rainwater, or snowmelt) associated with areas where commercial, industrial, storage, transport, or service activities are conducted. The composition of this wastewater will vary depending on the nature of the facilities’ activities in a given area. Municipal wastewater is understood as domestic wastewater or a mixture of domestic wastewater with industrial wastewater, rainwater, or snowmelt, which is discharged through the municipal sewer system [1].

The composition and quantity of wastewater are variable and depend on various factors, including the population size of a given region, the degree of industrialisation, and the extent of city sewerage. Additionally, the volume and type of generated waste are influenced by the legal regulations in a specific area and the season the wastewater is produced [4,5]. Essentially, wastewater consists of 99.94% water by weight, with the remaining 0,06% being dissolved or suspended contaminants [3]. Untreated wastewater may contain organic substances (proteins, carbohydrates, lipids, detergents) [6] and inorganic substances (heavy metals) [7], as well as various pathogenic microorganisms (dangerous viruses, bacteria) [8,9]. Additionally, wastewater may be contaminated with pharmacological agents (antibiotics, hormones) and may sometimes contain radionuclides [10,11,12].

### 1.1. Negative Impact of Wastewater

Due to the high biological harmfulness of most types of wastewater, it should be processed in wastewater treatment plants before being discharged into the environment. However, it is estimated that most developing countries (90%) discharge wastewater directly into ponds, lakes, seas, oceans, and rivers without any prior treatment. Such actions are detrimental to the environment and pose a threat to the health of both humans and animals [13,14]. Harmful compounds in untreated wastewater discharged into the environment can enter the food chain and cause numerous health problems, such as cancer, delayed nervous responses, and DNA mutations [15]. Additionally, wastewater effluents can infiltrate drinking water sources, leading to the spread of pathogens such as viruses (poliovirus, adenovirus, and norovirus) bacteria (*Escherichia coli*, *Salmonella* spp., *Campylobacter* spp., *Vibrio cholera*), protozoa (*Cryptosporidium* spp., *Giardia* spp.,) and helminths (*Ascaris* spp., *Trichuris* spp., tapeworms) [16].

Helminths are an artificial multicellular organism group that parasitises humans and animals. They are widespread, especially in developing countries and tropical and subtropical areas, leading to significant morbidity and economic losses in production. The WHO (World Health Organization) estimates that around 1.5 billion people suffer from helminth infections worldwide and emphasises that their high prevalence is associated with poor access to clean water [17]. Depending on the species, they may cause symptoms ranging from mild to severe. For example, pinworm infection is associated with itching around the anus and does not pose a serious health problem [18]; moreover, the infection can be easily controlled. On the other hand, *Schistosoma* spp. infection leads to 200,000 deaths annually [19], and its control is becoming more difficult due to the development of drug-resistant worm populations [20]. In the EU and the US, the infection rate is significantly lower due to higher sanitation standards and more severe climates. However, climate change, globalisation, and the ease of travel urge us to start seriously considering the efficiency of water treatment technologies in helminth control.

More and more countries are struggling with water shortages, often using wastewater that has not been fully treated to irrigate crops. Wastewater is characterised by a high content of nutrients such as phosphorus and nitrogen, which are essential for plant growth. However, when these elements enter aquatic ecosystems in excess, they can cause eutrophication [14,21,22]. The secondary use of untreated or partially treated wastewater also increases the risk of intestinal nematode and protozoan infections, such as *Ascaris lumbricoides*, *Trichuris trichiura*, *Giardia lamblia*, and *Cryptosporidium parvum*. These parasites can cause acute gastrointestinal disorders and chronic diseases, especially in children and young livestock, which are particularly vulnerable [23,24,25]. Furthermore, using byproducts generated during wastewater treatment, such as sewage sludge, may also raise controversies. Contaminated sewage sludge (e.g., with heavy metals or parasites) poses risks to public health and environmental safety. However, proper sewage sludge management, which includes monitoring and treatment processes, mitigates any issues associated with its uncontrolled release into the environment. In developed countries, such as the Member States of the European Union, regulations are in place to ensure the safe use of sewage sludge. The appropriate management of sludge poses no threat to humans, animals, or the environment and provides several benefits, including economic ones.

### 1.2. Benefits of Wastewater Treatment

As the global population increases, water demand also rises. Water is essential for food production, the cultivation of crops, and raising livestock, and it has a huge impact on demographic, economic, and military situations worldwide. Water is used in households on a daily basis [14]. However, freshwater resources are limited globally. According to data provided by UNICEF in 2022, 2.2 billion people worldwide still lacked access to safely managed water services [26]. The problem of insufficient access to clean water is often linked to the political and economic situation of a particular country, as well as to social factors such as a lack of education, bad hygiene habits, and low knowledge regarding waterborne pathogens. These factors contribute to the high death rate associated with contaminated water, which can be up to twice as high in low-income countries. Moreover, low-income countries spend limited financial resources on the construction of water treatment infrastructure, which further reduces access to clean water, resulting in social inequalities within a country, as the presence of the appropriate infrastructure may be restricted to more urbanised areas. Low-income countries also struggle with insufficient resources to counteract climate change, further reducing water availability. In countries facing water scarcity, implementing effective water policies should be essential [27]. One method of addressing the water scarcity problem is to reuse the wastewater. To ensure that this solution is safe for the environment and human and animal health, appropriate wastewater treatment and monitoring are crucial [15].

Treated wastewater can be used in agriculture, especially in countries facing water shortages, where it can be employed for irrigating crops. This solution also reduces the demand for groundwater extraction, providing economic benefits by requiring lower financial investments. Furthermore, treated wastewater is nutrient-rich (e.g., with N, P, K, Ca), enhancing crop yields [28,29,30,31]. During the wastewater treatment process, sewage sludge is also produced, which, after proper treatment, becomes a valuable source of nutrients and organic matter. Utilising wastewater and sewage sludge as fertiliser reduces the need for synthetic fertilisers in agriculture, lowering production costs. Additionally, sewage sludge can serve as the energy source for heat and electricity production. Therefore, reusing sewage sludge offers economic and environmental benefits [30,31].

## 2. Legal Acts

At the end of the 20th century, global interest in wastewater reuse increased due to its numerous benefits. However, the risks to public health and the environment prompted a series of guidelines concerning more efficient wastewater management [28]. The first WHO guidelines on the use of wastewater for agricultural and aquaculture purposes were developed in 1973, aiming to protect public health and facilitate the rational use of wastewater and excreta in these sectors. The document “Reuse of Effluents: Methods of Wastewater Treatment and Health Safeguards” was developed with the assumption of a low risk and without conducting epidemiological studies [32,33].

It was not until 1989 that updated guidelines were published, which included a thorough analysis of the available epidemiological studies. These guidelines focused primarily on protecting public health, emphasising minimising contact with pathogens and examining microbiological parameters (faecal coliforms and helminth eggs) in wastewater intended for irrigation. Another update of the WHO guidelines was published in 2006, considering the oversight of wastewater use and the importance of wastewater monitoring [28,33,34,35,36].

Around the same time, the FAO (Food and Agriculture Organization of the United Nations) also developed guidelines for the safe use of wastewater in agriculture. In 1987 and 1999, the FAO published guidelines on reducing water usage due to salinity, infiltration parameters, the toxicity of sodium, chloride, or boron, and the reuse of treated water in agriculture and treatment requirements [28].

The European Union also developed documents on wastewater reuse. One of the main acts is the Council Directive of 21 May 1991, concerning urban wastewater treatment [1]. The directive aims to protect the environment from the adverse effects of discharging inadequately treated wastewater. It imposes obligations on Member States regarding wastewater treatment and pollution reductions. It has been updated several times to align with the latest technological and ecological requirements [37].

Another significant legal act in the European Union is Regulation (EU) 2020/741 of the European Parliament and of the Council of 25 May 2020 on minimum requirements for water reuse. This document was created in response to the need to standardise and unify regulations on water reuse in different Member States. It aims, among other things, to protect the environment and public health and ensure the safety of reclaimed water in agriculture. The regulation introduced quality standards, water monitoring, and risk management procedures, setting minimum quality requirements for water reclaimed from treated wastewater [38].

The issue of sewage sludge contamination with pathogens, and consequently with parasite eggs, is indirectly regulated by Directive 86/278/EEC [39], which provides guidelines aimed at preventing the negative effects of using sewage sludge for agricultural purposes, thereby protecting the environment and public health. The directive obliges Member States to control the content of heavy metals and other chemical contaminants in sewage sludge. Although it does not directly address the presence of parasites and their eggs in sewage sludge, it allows Member States to establish additional, stricter national regulations concerning sludge quality, including the content of parasite eggs. For example, in Poland, the Regulation of the Minister of the Environment of 6 February 2015 on the use of municipal sewage sludge was introduced (O.J. 2015, item 257) [40]. This regulation provides detailed guidelines on the quality, application, monitoring, and management of municipal sewage sludge. It regulates the principles of safe sludge use to ensure public health and environmental protection. It also specifies the scope, frequency, and reference methods for testing sewage sludge and the soils where it is to be applied. The regulation includes acceptable concentrations of heavy metals and other contaminants (including bacteria and parasite eggs) in sewage sludge intended for use on agricultural, reclaimed, and non-agricultural land. It focuses in particular on *Ascaris* spp., *Trichuris* spp., and *Toxocara* spp. eggs. For example, it notes that for agricultural purposes, no eggs should be found in 1 kg of dry matter [40]. However, this Polish regulation does not account for all potential pathogens that may be present in wastewater sludge. In addition to helminth eggs (*Ascaris* spp., *Trichuris* spp., *Toxocara* spp.), other parasite species, such as *Echinococcus* spp., may also be present in sewage sludge [41]. Moreover, this legislation does not specify the methods to be used for detecting these parasites, which can lead to discrepancies in the results obtained by different laboratories.

Apart from these documents, other legal acts in the European Union impact wastewater regulations and its reuse. These include Directive 2000/60/EC of the European Parliament and of the Council of 23 October 2000, establishing a framework for Community action in the field of water policy [42]; Directive (EU) 2020/2184 of the European Parliament and of the Council of 16 December 2020, on the quality of water intended for human consumption [43]; the Council Directive of 12 December 1991, on the protection of surface waters against pollution caused by nitrates from agricultural sources [44]; the Council Directive of 12 June 1986, on the protection of the environment, and in particular of the soil, when sewage sludge is used in agriculture [39]; and Directive 2010/75/EU of the European Parliament and of the Council of 24 November 2010, on industrial emissions (integrated pollution prevention and control) [45]. The European Parliament and Council Regulation (EU) 2020/741 of 25 May 2020, on minimum requirements for water reuse, stipulates that reclaimed water used for irrigating pastures or fodder crops must contain no more than one helminth egg per litre [38]. Additionally, under the European Parliament and Council Directive (EU) 2020/2184 of 16 December 2020, water intended for human consumption must be free from any microorganisms and parasites [43].

## 3. Selected Parasites Found in Sewage Sludge

There are a number of relationships between organisms, such as mutualism, commensalism, predation, and parasitism. The broad definition of parasitism says that parasites are organisms that are harmful to their hosts and benefit from them. These parasites have adapted so as to colonise the host organism and have a negative impact on the host, leading to several negative outcomes such as disturbances in programmed cell growth, programmed cell death and cell division, diarrhoea, immunosuppression, allergies, the decreased efficiency of vaccines, and many other clinical symptoms. In addition, the parasite’s presence affects the host’s lifespan and fertility [46].

Various developmental stages of parasites are released into the environment and can be found in the water, sewage, or on vegetables and fruits. For example, *Taenia* spp., *Hymenolepis* spp., *Entamoeba* spp., *G. lamblia*, *Ascaris* spp., *T. trichiura*, and *Toxocara* spp. were found on vegetables from Soran City (Iraq) [47]. The number of detected parasites depends on factors such as the detection method used, the detection timeframe, or the sample’s origin [48]. In Sweden, a trend of increased numbers of parasitic protozoa in samples from wastewater treatment plants was observed from February to June [49]. Furthermore, in Tunisia, significantly more parasites were detected in raw sewage samples than in treated sewage samples [50]. The number of parasites detected in treated and raw sewage is particularly significant in arid regions where wastewater agricultural reuse is practised [51].

The average load of parasite eggs in Morocco in raw wastewater is about 9 eggs per litre. For the treated wastewater, this number is lower, and it is less than 1 egg per litre [51], but it also varies depending on the region in the city. Studies indicate that a greater number of detected parasites are recorded in Northern Africa. It was found that this continent has a higher average contamination with parasites in raw sewage [52]. However, it should be noted that factors such as hygiene standards or sewage treatment may differ between regions belonging to a given country or climate zone. These factors may affect the correlation between place and the number of detected parasites [53].

The appearance of parasites in sewage may affect human health and indicate emerging infection hotspots. Studies conducted in Spain revealed a high incidence of protozoal infections, which coincided with the rate of parasite detection [54]. Additionally, in Brazil, a reduction in parasite prevalence was associated with increased sewer coverage in the study areas [55].

**Figure 1:**
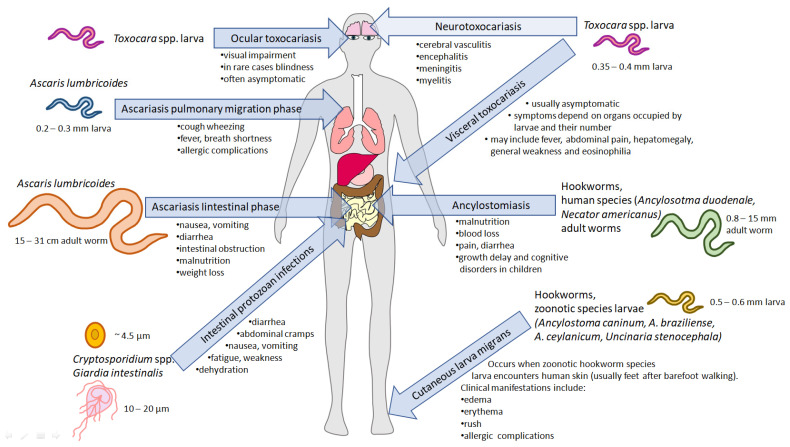
The most significant water, wastewater, and sewage-borne parasitic infections and their impact on humans. Parasites living in the gastrointestinal tract release eggs, cysts (*Giardia intestinalis*), or oocysts (*Cryptosporidium* spp.) through faeces into the environment. The life stages undergo various transformations, leading to the development of the infectious stage. *Toxocara* spp. (*canis* or *cati*) eggs are released by dogs or cats, respectively, and may be ingested by humans. In the intestine, larvae hatch but do not develop into adult worms. Instead, they migrate to different tissues, leading to diseases depending on the infected organ: ocular toxocariasis, visceral toxocariasis, and neurotoxocariasis. *Ascaris lumbricoides* larvae (blue) hatch in the intestine and migrate to the lungs, leading to clinical complications. After the lung stage, the worms migrate back to the intestine, where they mature, causing ascariasis. The course of hookworm infection varies with the species. Human species (green), such as *Ancylostoma duodenale* and *Necator americanus*, penetrate the skin to infect and mature into adult forms in the intestine, leading to ancylostomiasis. Zoonotic species (yellow) larvae, upon contact with human skin, cannot complete the life cycle and migrate through the skin, resulting in serious complications. Protozoan parasites, such as *Cryptosporidium* spp. (orange) and *Giardia intestinalis* (grey) may also inhabit the gastrointestinal tract, and the infection occurs upon ingesting their infective forms.

Several pieces of literature data deal with the controversial issue of *Ascaris suum* and *A. lumbricoides*. There is a theory stating that these two species are actually one species, so some studies indicate the possibility of synonymising these two species [56]. This is due to their high morphological similarity, the possibility of cross-infection and interbreeding [57,58], and the similarity of their mitochondrial genome structures [59]. However, some studies indicate genetic diversity between the studied populations [60]. The *A. suum* genome has been sequenced, producing a draft assembly of 272,782,664 base pairs and 18,542 predicted genes. SNPs (single nucleotide polymorphisms) were also found within the coding regions of the genome, and a high nucleotide variability was noted. In addition, the potential for intervention was found within the least variable genes, which encoded *i.a* receptors or threonine–serine phosphatases [61]. The regions used to identify *A. lumbricoides* and *A. suum* were ITS1, ITS2, and the *cox1* gene [62]. Another important issue was to identify SNPs located in the β-tubulin gene family that would contribute to resistance to benzimidazoles [63]. *A. lumbricoides* may lead to two types of pathology. The first is associated with an immune response against migrating larvae, which are likely to cause eosinophilic pneumonia associated with shortness of breath, cough, fever, or tender hepatomegaly [64]. *Ascaris* spp. entering the lungs (Figure 1) can be particularly dangerous for children [65]. Due to the migration of larvae, hepatobiliary ascariasis can also develop in the host. This type of ascariasis causes biliary colic, acute cholangitis, acute cholecystitis, or liver abscesses [66]. Adult roundworms cause nutrient depletion along with gastrointestinal obstruction. The infection is often asymptomatic, but abdominal pain, intestinal volvulus, and intussusception may occur [64]. *Ascaris* spp. infection can cause complications such as intestinal perforation [67]. The problem associated with roundworm eggs is related to their durability and resistance to external factors, allowing for survival in soil for up to 7 years [68]. Both acetic acid and ammonia have been proven to be effective against *A. lumbricoides/suum* eggs, which can be found, for example, on vegetables. However, the concentration of acetic acid had to be much higher in this case due to the durability of the eggs [69]. Still, the efficient concentration of ammonia may be encountered in sludge [68].

Other *Ascaridoidea* superfamily members that are a serious threat to public health are two species of roundworms, *Toxocara canis* and *Toxocara cati* [70], which develop into adults (living in the intestine) in their definitive hosts: dogs and cats, respectively. Upon the infection of paratenic hosts (i.e., humans), their development is significantly different. Upon the hatching of the eggs in the intestine, the larvae migrate towards various tissues (Figure 1) [71], and the clinical symptoms of the disease are associated with the occupancy of the particular organ. Four clinical classes of toxocariasis may be distinguished: visceral larva migrans, neurological toxocariasis, ocular larva migrans, and latent toxocariasis [72]. Ocular larva syndrome may be associated with blindness [73]. Symptoms of neurological toxocariasis may include epilepsy [74], meningitis, encephalitis, cerebellar vessels, or optic neuritis [75]. In addition to the common symptoms, novel research shows that *Toxocara* spp. infection may have other long-term negative effects. Dogs that were infected have reduced numbers of intestinal flora, and their composition is similar to that of the flora carried by the parasite [76]. *T. canis* may be a potential contributor to Alzheimer’s disease due to its involvement in disrupting cholesterol homeostasis, which can lead to neurodegeneration. This disruption, in conjunction with alterations in the amyloid pathway, suggests a link to the development of Alzheimer’s disease [77]. Molecular identification may also be associated with genomic or mitochondrial DNA detection. In the draft genome of *T. canis*, the total length of the genome was determined to be 341,776,187 base pairs, and it contained 20,178 genes [78]. Moreover, the possibility of 715 essential homologues was also found. These include ion channels, transporters, kinases, peptidases, and phosphatases. Some of the essential effector genes were associated with the RNAi pathway. In addition, the *sid-1* gene, present in *A. suum*, was found in *T. canis* [79]. In studies attempting to distinguish *T. canis* from other species belonging to the genus *Toxocara* and to investigate the evolutionary relationships of the organisms, the ITS-2 region, the *rrnL* [80], and *cox1* genes were used [81]. Furthermore, a nested multiplex PCR (polymerase chain reaction) assay was developed to detect the infection. This assay could distinguish between *T. canis*, *T. cati*, and *A. suum*. It was also found that *A. suum* and *A. lumbricoides* could not be distinguished using this system [82]. *Toxocara* spp. eggs, similar to *Ascaris* spp. eggs, may persist in the environment for a long time, which, in association with difficulties in molecular identification, requires the development of novel techniques allowing for the rapid detection of the roundworms in sewage and water.

Another group of soil-transmitted helminths are hookworms (nematodes from *Ancylostomatidae* family): *Ancylostoma duodenale*, *Ancylostoma ceylanicum*, *Ancylostoma caninum*, and *Necator americanus* [70]. Human infection can lead to blood loss from the intestine, resulting in iron deficiency, hypoproteinaemia, anarca, weight loss, vomiting, anaemia, and eosinophilia [83]. Larvae hatch from eggs within 1–2 days, and L3 larvae can stay in the environment for up to a month under appropriate conditions. In addition, these parasites can stay for years in the human small intestine [84]. In contrast to *Ascaris* spp., the invasive form of hookworms is L3 larvae not protected by the egg shield, making them more prone to the technological and chemical processes engaged in sewage treatment.

Furthermore, dangerous parasites directly detected in wastewater [41] include individuals from the genus *Echinococcus* (family Taeniidae). Species whose eggs can cause human infection are *Echinococcus granulosus*, *Echinococcus multilocularis*, *Echinococcus vogeli*, and *Echinococcus oligarthrus*. Following development in a host, larvae or metacestodes cause various echinococcoses depending on the species. Human echinococcoses include cystic, polycystic, unicystic, and alveolar echinococcosis [85,86]. Moreover, the geographical distribution of these echinococcoses varies [87]. For example, in Poland, hydatid disease (echinococcosis) is observed, with *E. granulosus* causing cystic echinococcosis and *E. multilocularis* causing alveolar echinococcosis. Moreover, the number of infections in Poland was most numerous in 2023 compared to the previous three years. Echinococcosis often does not show symptoms but can be particularly dangerous if parasites develop within the central nervous system or the eye [88]. Symptoms of cystic echinococcosis may include fever, pain, jaundice, haemoptysis, and tumour formation. The complications observed in patients include mechanical (fistulas, cyst rupture), immune–allergic (anaphylaxis), infectious (coinfections with bacteria and fungi), and mixed types [89]. The treatment of hydatid disease involves the surgical removal of cysts and drug therapy [88]. Individuals of this genus have been detected in wastewater [41] and on vegetables [90]. Survival of *E. multilocularis* eggs in the environment has been observed for over 100 days. Additionally, their viability and infectivity were noted after 240 days of being suspended in tap water [91]. Furthermore, the survival and infectivity of eggs were observed under varying low and high temperatures [92], as well as after exposure to disinfectants [91].

## 4. Methods for Identifying Parasites in Wastewater

Various microscopic and molecular methods have been developed for detecting parasites (Table 1). Numerous studies identify parasite eggs in wastewater and sewage sludge. For instance, in 2016, research conducted in wastewater from Puno, Peru, confirmed the presence of parasite eggs using microscopic observations [93]. Helminth eggs have also been detected in wastewater from Iran, India, and Morocco [94,95,96]. Bastos et al. demonstrated the presence of parasite eggs in sewage sludge [97]. However, identifying parasite eggs through microscopic techniques is time-consuming, requires expertise, and cannot be automated. Moreover, artefacts and flocculants used to form larger particles in sludge hinder microscopic diagnostic [98]. To address these challenges, a digital imaging system for identifying and quantifying several helminth egg species in wastewater was developed in 2016 (*T. trichiura*, *T. canis*, *A. lumbricoides*, *Taenia saginata*, *Hymenolepis nana*, *Hymenolepis diminuta*, *Schistosoma mansoni*). This system provides information on the number of eggs per species and counts the total egg quantity, distinguishing whether the detected *A. lumbricoides* eggs are fertilised. The system’s specificity is around 99%, with a sensitivity ranging from 80 to 90% [99].

Molecular techniques are also used to detect parasites in wastewater and sewage sludge. Compared to microscopy-based methods, PCR offers a higher specificity and sensitivity, thus facilitating parasite species identification in wastewater and sewage sludge [14]. For instance, in 2015, a quantitative PCR (qPCR) method was developed to detect *A. caninum* eggs in wastewater, with a detection limit of 500 fg gDNA [100]. In 2017, a study using the qPCR method assessed the prevalence of intestinal parasitic protozoa (*Cryptosporidium* spp., *Giardia intestinalis*, *Entamoeba histolytica*, *Entamoeba dispar*, *Dientamoeba fragilis*) in Swedish wastewater [49]. In 2020, a study detected the presence of intestinal parasitic protozoa and nematodes in wastewater samples from Spain using qPCR for *Cryptosporidium* spp., *Giardia duodenalis*, and *Entamoeba* spp., while nematode eggs were detected using optical microscopy [54]. The limitation of PCR and qPCR methods is their inability to distinguish between live and dead parasite eggs. This issue can be addressed by using the DNA intercalating dye propidium monoazide (PMA) in combination with PCR and qPCR methods. During photoactivation, PMA penetrates dead eggs, forming a stable DNA-PMA complex that prevents DNA amplification during PCR. In 2016, a study described the PMA-qPCR method, confirming its suitability for selectively detecting live helminth eggs in environmental samples, including wastewater. However, this method also has limitations similar to the viability staining methods, as it depends on the structural integrity of live and dead parasite eggs. False results may occur when assessing recently inactivated eggs, as they require 12 h of incubation to become permeable. Factors such as dye concentration, light exposure, and incubation time also influence this method’s effectiveness [101,108]. Many of the methods indicate the viability of eggs without considering their invasiveness. These two statements are not synonymous, and the use of conditions such as low temperatures and the aerobic and anaerobic digestion of sewage sludge may result in the eggs being viable but their invasiveness being reduced [102].

Multiplex-based PCR approaches allow for the rapid and simultaneous amplification of multiple parasitic species in a single sample. A multiplex quantitative (qPCR) test has been described for detecting helminths such as *A. lumbricoides*, *N. americanus*, *Ancylostoma* spp., *T. saginata*, and *Taenia solium* in faeces [103]. A nested multiplex PCR test for detecting *T. canis*, *T. cati*, and *A. suum* has also been developed, although it was designed to detect these parasites in meat and offal. The detection limit of this test was 10 fg of genomic DNA for *T. canis*, 1 fg for *T. cati*, and 100 fg for *A. suum* [82]. PCR-based methods are sufficiently specific, sensitive, and efficient for assessing the presence of parasite eggs in wastewater and sewage sludge. However, these analyses require professional equipment and trained personnel. In many endemic countries, access to such resources is limited, potentially leading to unreliable results. In 2020, the RPA-LF (recombinase polymerase amplification combined with lateral flow strips) test was developed to detect helminth eggs. This test was created as a rapid, sensitive, highly specific, and cost-effective alternative to PCR. Additionally, the equipment required for this method is portable, which allows the analysis to be performed directly at wastewater treatment plants. This method can detect 2 fg of gDNA, indicating its potential to detect even a single helminth egg in a sample. Using a multiplex approach, a single lateral flow strip successfully detected eggs of two different helminth species [109]. A method utilising digital droplet PCR (ddPCR) has also been described for detecting parasitic protozoa in wastewater, showing high sensitivity, with a detection limit of 1.32 copies per 20 μL reaction volume for *C. parvum* [104].

Several studies have used the next-generation sequencing (NGS) technique to assess wastewater composition, which may further facilitate the detection of helminths in sewage. In 2019, a study used NGS to identify eukaryotic microorganisms in wastewater samples from four Australian wastewater treatment plants at different treatment stages, detecting human intestinal parasites [110]. In 2020, NGS based on amplicon sequencing was used to detect and differentiate intestinal parasitic protists in wastewater samples from Swedish treatment plants, identifying species such as *Blastocystis* sp., *Entamoeba moshkovskii*, *E. histolytica*, *E. dispar*, *Entamoeba hartmanni*, *Endolimax nana*, and *Iodamoeba bütschlii* [105]. In 2022, a study evaluated the entire wastewater microbiome using shotgun metagenomic and metatranscriptomic sequencing of wastewater samples from Switzerland [111].

## 5. Viability vs. Infectivity

The viability of a parasite egg is its ability to survive and to develop further. Viability can be influenced by environmental factors such as temperature [112], pH, dryness, egg developmental stage, and parasite species [113]. Specific environmental conditions can render eggs non-viable, making it crucial to have methods for the assessment of eggs’ viability. For testing the viability of oncospheres after egg hatching, trypan blue staining can be used. In this method, live oncospheres do not change colour, whereas dead ones exhibit a colour change under a microscope [114]. Another staining technique used to determine egg viability involved the LIVE/DEAD BacLight Bacterial Viability Kit, type 7007 (Molecular Probes, Invitrogen, Eugene, OR, USA). In these studies, live eggs of *Toxocara* spp., *Ascaris* spp., and *Trichuris* spp. were stained green or green-blue, while dead cells appeared red [115]. Other dyes used for viability assessment include methylene blue [116] and eosin solution [117].

In addition to staining methods, microscopic observation methods are also used to determine eggs’ viability. For samples containing *A. suum* eggs, a 1% sodium hypochlorite solution was added, and the samples were observed under a microscope. Live eggs were identified by the presence of internal structures (two or more defined cells) or motile larvae [118]. Some studies combine microscopic observation with dye application, such as trypan blue, to monitor egg hatching [113]. Microscopic observation can also focus solely on the larval stage of parasites obtained after prior egg hatching [119]. Additionally, worm viability studies using microscopy allow one to assess the movement of worms. Before determining non-viability in samples where no movement is observed, the worms are exposed to light and/or shaken vigorously [120]. It is important to consider the varied sensitivity of tests. For example, differences in the sensitivity of certain tests (among others, SYTO-9 assays and the mouse infectivity assay) used to study the inactivation of protozoan cysts after ozone treatment were observed [121].

Parasites’ infectivity is their ability to infect a host. It is important to note that viability is not necessarily linked with high invasiveness. For example, eggs lose their infectivity with extended egg storage time [122]. The use of therapeutic agents is another factor influencing infectivity. For instance, larvae treated with ivermectin and albendazole were not infective to mice [123]. Egg infectivity can be determined by administering them to animals and then collecting and observing samples from these animals using an appropriate method [118,123]. However, these methods are very time-consuming [118] and require the cost of maintaining the animals used in the study, making them unsuitable for routine sewage examination. Due to those limitations, the development of new methods to assess the potential of helminth eggs to infect the host could be a reasonable solution.

By determining the parameters that cause a loss of viability, we can identify ways to eliminate eggs. Egg inactivation often involves using a specific combination of several parameters (pH, temperature, dryness) [113]. Environmental conditions can also affect eggs’ viability. For example, temperature, salinity, and light influence *Anisakis simplex* eggs’ hatching ability and hatched larvae survival [124]. In contrast, the activity of *Taenia* spp. is influenced by factors such as UV light, temperature, and lime [53]. Factors that inactivated *Pseudocapillaria tomentosa* eggs included UV light, chlorine, or dehydration [125]. The elimination of parasite eggs from wastewater and sewage sludge is also important. Sludge dewatering on drying beds results in the elimination of *A. duodenale* eggs from the sludge. Similarly, the co-composting of the primary sludge with date palm waste for 60 days resulted in the elimination of *Ascaris* spp. eggs with 98% effectiveness [106]. The impact of using urea to inactivate *Ascaris* spp. eggs has also been investigated [126]. Moreover, the use of the aerobic and anaerobic fermentation of sewage sludge reduced egg invasiveness even though they were viable [102]. Applying appropriate conditions during wastewater and sludge treatment can significantly reduce the viability or invasiveness of eggs. Factors that may affect the eggs’ viability during wastewater treatment include exposure time, the use of ammonia [127], and lime (pH/temperature) [128]. Specific temperature, pH, and dryness conditions affected the activity of *A. lumbricoides*, *A. suum*, *T. canis*, *T. trichiura*, *H. nana*, and *T. solium* [113]. The application of lime [129] and ammonia [127] resulted in the reduced viability of *A. suum*, but the use of disinfectants did not alter the infectivity of *E. multilocularis* eggs [91]. However, the application of *Daphnia pulicaria* affected the viability and infectivity of two protozoa species (*Giardia lamblia*, *Cryptosporidium parvum*) cysts [130]. Additionally, *C. parvum* exhibited decreased viability and infectivity following ozonation [121].

## 6. Conclusions

Parasites are a global health burden, especially in tropical regions and low-income countries. They are a problem in wastewater management and the reuse of sludge as fertiliser and for other purposes. Although some existing legal regulations prohibit the use of sludge containing parasite eggs as fertiliser, the presence of eggs may not always pose a danger due to the loss of their infectivity. Nonetheless, to ensure the effective management of wastewater treatment byproducts such as sewage sludge, it is essential to establish appropriate standards for monitoring the presence of parasites and assessing their infective potential. Developing such standards would improve the reproducibility, quality, and accuracy of results across various laboratories. Another avenue for improving wastewater diagnostics is to assess not only the presence of the eggs but also the invasiveness of the eggs. Implementing such an approach could lead to increased sensitivity and changes in restrictions on using sewage sludge as fertiliser when eggs are present but show no viability.

## Figures and Tables

**Table 1:** Comparison of methods detecting the presence of parasites.

Sample Type	Used Method	Detected Parasites	Country	Comments
Influent and effluent wastewater [49]	Multiplex real-time PCR	*Giardia intestinalis* *Entamoeba dispar* *Dientamoeba fragilis*	Sweden	The tested treatment plants used mechanical, chemical, and biological treatment. Two multiplex real-time PCR reactions were performed, the first one specific for *Entamoeba histolytica*, *E. dispar,* and *D. fragilis*, and the second one for *Cryptosporidium parvum/hominis* and *G. intestinalis.*
Raw and treated wastewater [50]	Microscopy	*Ascaris* sp. *Entamoeba coli* *Entamoeba histolytica/dispar* *Enterobius vermicularis* *Giardia* sp. *Hymenolepis nana* *Taenia* sp.	Tunisia	The studied treatment plants used activated sludge treatment and waste stabilisation ponds. A modified Bailenger method was used to determine the presence of parasites.
Raw and treated wastewater [51]	Microscopy	*Ascaris* sp. *Toxocara* sp. *Capillaria* sp. *Hymenolepis nana* *Hymenolepis diminuta* *Spirometra* spp.	Morocco	The tested treatment plants used natural lagoons during treatment. The concentration of parasite eggs dispersed in a biological sample was assessed using the Arther–Fitzgerald technique.
Samples of influent and effluent wastewater and selected intermediate stages of wastewater treatment [54]	Optic microscopy and PCR techniques	*Cryptosporidium* spp. (*Cryptosporidium hominis*, *Cryptosporidium parvum*) *Giardia duodenalis* *Entamoeba histolytica* *Entamoeba moshkovskii* *Entamoeba dispar*	Spain	PCR techniques were performed to identify the presence of *Crysptosporidium* spp., *Giardia duodenalis*, and *Entamoeba* spp. Molecular techniques proved to be more sensitive in detecting parasites and allowed one to distinguish between the practically identical morphological species of *Entamoeba histolytica*/*dispar*/*moshkovskii.*
Mouse or chicken liver [82]	Nested multiplex PCR	*Toxocara canis* *Toxocara cati* *Ascaris suum*	Japan	Both mouse and chicken livers were infected with parasites isolated from dogs (*Toxocara canis*), cats (*Toxocara cati*), and pigs (*Ascaris suum*). Multiplex PCR has been shown to be much more sensitive than the direct counting of larvae after tissue digestion.
Fresh domestic wastewater–influent and effluent in up-flow Anaerobic Sludge Blanket laboratory reactor [93]	Optic microscopy	*Ascaris lumbricoides* *Toxocara* spp. *Hymenoloepis nana* *Enterobious vermicularis*	Peru	
Raw and treated wastewater [94]	Microscopy	*Ascaris lumbricoides* *Trichostrongylus* spp. *Enterobius vermicularis* *Ancylostoma duodenale* *Necator americanus* *Taenia* spp. *Hymenolepis nana* *Dicrocoelium dendriticum*	Iran	In the treatment plants, activated sludge or stabilisation pond treatment was used. A modified Bailenger’s method was used.
Faecal sludge or fresh wastewater samples [95]	Microscopy	*Ascaris* sp. *Trichuris* sp. hookworm *Hymenolepis nana* *Hymenolepis diminuta* *Aspiculuris* sp. *Heterakis spumosa* *Trichosomoides crassicauda* *Calodium hepaticum* *Capilaria hepatica*	India	Faecal sludge was collected from desludging trucks, and fresh sewage was collected from an apartment complex and a shared toilet.
Raw, decanted, treated wastewater [96]	Microscopy	*Giardia lamblia* *Entamoeba histolytica* *Entamoeba coli* *Strongles* sp. *Ancylostoma* sp. *Enterobius vermicularis* *Ascaris* sp. *Tichuris trichiura* *Capliaria* sp. *Fasciola hepatica* *Taenias* sp. *Hymenolepis nana* *Hymenolepis diminuta*	Morocco	The treatment plants used anaerobic lagoons, infiltration–percolation, and, additionally, UV radiation in one of the treatment plants. Bailenger’s method was used for the parasitological analysis.
Sewage sludge [97]	Microscopy	*Ascaris* sp. *Capillaria* sp. *Enterobius vermicularis* *Fasciola hepatica* *Hymenolepis* sp. *Taenia* sp. *Toxocara* sp. *Trichuris* sp.	Brazil	The activated sludge treatment method was used in the sewage treatment plants. The average precision of the method used in the tested samples was 26.3%.
Sewage sludge [98]	Microscopy	*Toxocara* sp. *Ascaris* sp. *Trichuris* sp.	Poland	The treatment plants used mechanical–biological wastewater treatment. The method used to analyse parasites involved the use of polyelectrolytes. A viability assessment was performed after the incubation of the eggs in a moist chamber at a temperature of approximately 27 °C.
Wastewater, sludge, and excreta processed at the laboratory [99]	Digital imaging system for identifying and quantifying selected parasites	*Ascaris lumbricoides* *Taenia saginata* *Toxocara canis* *Trichuris trichiura* *Hymenolepis nana* *Hymenolepis diminuta* *Schistosoma mansoni*		Three versions of the system were developed to increase the specificity and sensitivity of the method.The system was adapted to detect parasite eggs, not larvae.
Tap water, secondary treated and raw wastewater, and sludge samples [100]	Real-time PCR	*Ancylostoma caninum*	Australia	The performed real-time PCR was directed against *Ancylostoma caninum*.
Raw wastewater, human faeces, and soil [101]	PMA-qPCR	*Necator americanus* *Ascaris lumbricoides*	Australia	The samples containing viable eggs were human faeces and soil samples.
Sludge [102]	Microscopy	*Toxocara canis* *Trichuris vulpis* *Trichuris suis* *Ascaris suum* *Hymenolepis diminuta*		Eggs were added daily to the research samples, which were added to laboratory aerobic and anaerobic bench-top digestors. During the experiment, the eggs’ viability was checked after applying various factors.
Human faeces [103]	Multiplex quantitative PCR	*Ancylostoma duodenale* *Ascaris lumbricoides* *Taenia saginata*	Philippines	The first PCR reaction performed was specific for *Ascaris lumbricoides*, *Ancylostoma duodenale*, *Necator americanus*, and *Taenia* spp. Samples positive for *Taenia* spp. were subjected to a specific reaction for *Taenia solium* and *Taenia saginata*.
Influent wastewater samples [104]	Droplet digital PCR	*Cryptosporidium parvum*		Optimisation of the ddPCR was performed, and the limit of detection for *Cryptosporidium parvum* using this method was determined to be 0.07 copies/μL (1.32 copies in a 20 μL reaction). A comparison of DNA isolation methods from *Cryptosporidium parvum* oocysts was conducted, revealing differences.
Influent wastewater [105]	ILLUMINA sequencing	*Entamoeba moshkovskii* *Entamoeba coli* *Entamoeba dispar* *Entamoeba hartmanni* *Entamoeba histolytica* *Endolimax nana* *Iodamoeba bütschlii* *Blastocystis* sp. *Entamoeba histolytica*	Sweden	
Dewatered and thickened primary sludge [106]	Microscopy	*Ascaris lombricoide* *Ancylostome duodenale* *Trichuris trichiura* *Capilaria* spp. *Hymenolepis nana* *Taenia saginata*	Morocco	The viability of *Ascaris* eggs was tested during the co-composting of dewatered primary sludge with date palm waste.
Wastewater before treatment [41]	Nested PCR Real-time PCR LAMP (loop-mediated isothermal amplification)	*Echinococcus multilocularis*	China	Using these methods, it was possible to detect *Echinococcus multilocularis* DNA in samples containing 20 eggs/L. The effectiveness of all the methods used in the study for detecting *Echinococcus multilocularis* was confirmed.
Stool [107]	Modified Ziehl–Neelsen stain and Giemsa stain	*Cryptosporidium parvum* *Blastocystis hominis* *Isospora* *Cyclospora caytenensis* *Entamoeba histolytica* *Giardia lamblia* *A. lumbricoides* *Taenia* sp. *Entrobious vermicularis* *Hymenolepis nana* *Strongyloides stercoralis*	Egypt	
